# The impact of circular stapler size on the risk of anastomotic stricture following total mesorectal excision in rectal cancer patients: A retrospective cross‐sectional study

**DOI:** 10.1002/hsr2.1658

**Published:** 2023-10-24

**Authors:** Behnam Behboudi, Seyed‐Mohsen Ahmadi‐Tafti, Seyyed‐Alireza Hosseini, Kiana Tadbir‐Vajargah, Mohammad Sadegh Fazeli, Alireza Hadizadeh, Amirhossein Poopak, Mohammad Reza Keramati, Alireza Kazemeini, Aryan Ayati, Hannaneh Yousefi‐Koma

**Affiliations:** ^1^ Colorectal Research Center Imam Khomeini Hospital Complex, Tehran University of Medical Sciences Tehran Iran; ^2^ Division of Colorectal surgery, Department of surgery Tehran University of Medical Sciences Tehran Iran; ^3^ Research Center for Advanced Technologies in Cardiovascular Medicine Cardiovascular Disease Research Institute, Tehran University of Medical Sciences Tehran Iran

**Keywords:** anastomotic leakage, anastomotic stricture, colorectal cancer, low anterior resection

## Abstract

**Introduction:**

Colorectal cancer (CRC) surgery complications are a major issue affecting morbidity and mortality rates. Anastomotic stricture, which occurs in almost 30% of patients after surgery for rectal cancer, is one of the most serious but underreported side effects. In this study, we tried to assess the effect of stapler size on anastomotic stricture rate.

**Materials and Methods:**

At our facility, all patients underwent low anterior resections (LAR) performed using an open laparotomy technique. A contour‐curved stapler and an end‐to‐end anastomosis (EEA) circular stapler were used in the double stapling technique (DST). All patients also underwent a protective loop ileostomy. Patients who developed stricture following leakage were excluded.

**Results:**

This study comprised a total of 173 rectal cancer patients. A 29‐mm circle stapler was used to anastomose 77 patients (44.5%), while a 31‐mm circular stapler was used to anastomose 96 patients (55.5%). Six individuals experienced strictures; two had a 29 mm stamper and four (4.4%) had a 31 mm one. There was no significant difference between the two groups (p:0.575). On aggregate, 8 patients experienced leakage; 3 (3.8%) of these patients received treatment with a 29 mm stapler, whereas 5 (5.2%) received treatment with a 31 mm stapler.

**Conclusion:**

this study found no statistically significant difference in the stricture rates and stapler size. The findings of this study provide credibility to the notion that in rectal cancer patients having LAR, strictures can be safely avoided by performing the anastomoses with both staplers.

## 1 INTRODUCTION

Complications following colorectal cancer (CRC) surgery are a major concern, contributing to an increase in early and long‐term morbidity and mortality. One of the most significant but underreported complications following rectal cancer surgery is anastomotic stricture, affecting up to 30% of patients.^1^, ^2^ Patients with anastomotic strictures without a diversion ileostomy may present with abdominal pain, altered bowel habits, faecal incontinence, and bleeding. These post‐stricture symptoms can negatively affect patients’ quality of life.^3^ However, in patients with a diversion ileostomy, anastomotic strictures are typically an incidental finding during a control colonoscopy. The abovementioned complaints are generally not reported in this subset of patients. Although novel treatment targets such as molecules in ncRNA–miR–mRNA network in response to Lactobacillus acidophilus are promising, surgery is still the gold standard in treatment; hence it is important to consider the complications.

Causes of colorectal cancers included, change of normal flora into pathogens in patients who have immunodeficiency due to the use of immunosuppressive drugs and role of some bacteria to producing gastrointestinal cancer.^4^, ^5^, ^6^ Several risk factors for the development of anastomotic strictures have been proposed, including neoadjuvant chemoradiotherapy, tissue ischemia, inflammation,^3^, ^7^ anastomotic leakage,^8^, ^9^ protective stoma,^8^, ^9^ stapled anastomosis,^3^, ^10^ and surgical technique.^2^ Moreover, recent studies have implicated stapler size in developing anastomotic strictures in CRC surgery.^7^, ^11^

Upper GI tract surgery studies on the relationship between stapler size and anastomotic strictures observed that a larger circular stapler was associated with a lower risk of anastomotic stricture.^12^, ^13^ In lower GI tract surgery, the stapler size in ileal pouch‐anal anastomoses (IPAA) has been studied, revealing that smaller stapler sizes are associated with higher but insignificant stricture rates. In rectal cancer surgery, these studies are scarce, yielding inconsistent results.^7^, ^8^, ^9^, ^11^ Considering the prevalence and clinical significance of anastomosis‐related complications, identifying risk factors associated with anastomotic outcomes seems necessary. Consequently, we aimed to investigate the association between circular stapler size and anastomotic stricture in patients undergoing surgery for rectal cancer at Imam Khomeini Hospital Complex in Tehran, Iran.

## 2 METHODS

This prospective cohort study was conducted at Imam Khomeini Hospital Complex in Tehran, Iran, a tertiary referral center. Our study included 173 histopathologically proven rectal adenocarcinoma patients who underwent elective total mesorectal excision (TME) between March 2017 and March 2021. All participants were treated with neoadjuvant chemoradiotherapy based on tumor staging (T3< or N ≤ 1). This study was approved by the Research Deputy and the Ethics Committee of the Tehran University of Medical Sciences (Reference number: IR.TUMS.IKHC.REC.1398.164) and was carried out per the ethical standards outlined in the 1964 Declaration of Helsinki and all subsequent revisions. All participants signed a written informed consent form. This manuscript has been prepared according to STROBE and SAMPL guidelines for clinical study reports.

All patients in this study underwent low anterior resection (LAR) via an open laparotomy approach. The surgical team consisted of three board‐certified colorectal surgeons with extensive experience. All patients had a high ligation of the inferior mesenteric artery and vein using the same technique. At the surgeon's discretion, the splenic flexure was mobilized to achieve a tension‐free anastomosis, which was created between the descending colon and rectum. A double stapling technique (DST) was performed using a contour‐curved stapler and an end‐to‐end anastomosis (EEA) circular stapler. The size of the EEA circular stapler was determined by the preferences of the chief surgeon based on the patient's condition and intraoperative factors. An anastomotic air leakage test was conducted if the “doughnuts” were incomplete. If positive, either anastomosis via the stapling technique or protective ileostomy was performed.

Furthermore, a protective loop ileostomy was performed in all patients. One month and 3 months after discharge, coloproctological outpatient follow‐ups including symptom assessment, rectal examination, and rigid proctoscopy were scheduled. Patients who exhibited stenosis during clinical examination and experienced the start of symptoms directly connected to this condition were referred for endoscopic dilatation. Before the cessation of a temporary ileostomy, a barium enema was performed to assess the presence of colonic anastomotic stenosis. In the event that a notable stenosis was detected, the procedure of endoscopic dilatation was carried out. The colonoscopy section of our institution's colorectal laboratory reexamined patients 6 months after the operation. One of the three members of our surgical team performed rectosigmoidoscopy on each patient using a 12.3 mm flexible recto sigmoidoscope. Anastomotic stenosis was characterized by the inability to insert a 12‐mm proctoscope through the anastomosis. The anastomosis distance from the anal verge was measured in all patients. The individuals who had anastomotic stenosis were subjected to endoscopic dilatation using metal bougie dilators of progressively larger sizes (Savary‐Gilliard dilators measuring 10 mm, 21 mm, and 24 mm, manufactured by Cook Medical, located in Winston‐Salem, NC). they were followed after the dilation in 6 and 12 months intervals.

The primary outcome measure was the subjective evidence of anastomotic stricture, defined as the failure in traversing the anastomosis site with the flexible recto sigmoidoscope.

Exclusion criteria of the study included: (1) Patients who had not received preoperative chemoradiotherapy, (2) Patients who were not candidates for curative‐intent surgical resection, (3) Patients for whom a complete resection (R0) was not possible, (4) Patients with tumor recurrence diagnosed by clinical, radiological, or endoscopic assessment (presence of sinus tract at the anastomotic site, (5) All patients who developed stricture following leakage. 6) all patinets with a history of cerebrovascular accidents, Inflammatory bowel diseases or uncontrolled Diabetes mellitus and hypertension were excluded in this study.

Data regarding the demographic and preoperative characteristics were retrospectively collected from the medical records. Each patient was assigned a code, and all data analyses were performed anonymously.

Our institution's most frequently used EEA circular staplers are the 29‐ and 31‐mm staplers. Participants were stratified according to stapler head size into 29‐ or 31‐mm cohorts. Age, gender, and the presence of anastomotic stricture were compared between study cohorts. In addition, we compared the characteristics of patients with and without anastomotic strictures.

### 2.1 Statistical analysis

We reported continuous data as mean ± standard deviation (SD), or median, interquartile range (IQR), for normally or non‐normally distributed data, respectively. Categorical data were expressed as frequency (percentage). In addition, the chi‐square test or Fisher's exact test was employed to determine the relationship between categorical variables. Furthermore, independent sample *t*‐tests and Mann–Whitney non‐parametric tests (for non‐normally distributed data) were used to compare the means of continuous variables with non‐normally distributed data. All statistical analyses were conducted using the IBM Statistical Package for Social Sciences software (SPSS, version 26). *P*‐values less than 0.05 were considered statistically significant.

## 3 RESULTS

A total of 173 rectal cancer patients from March 2017 to March 2021 were included in this study. All patients had undergone neoadjuvant chemoradiotherapy, followed by elective surgical resection.

Seventy‐seven patients were anastomosed with a 29‐mm circular stapler (44.5%), and 96 patients were anastomosed with a 31‐mm circular stapler (55.5%).

Eighty‐one patients were female (46.8%), and 92 (53.2%) were male. Forty‐nine female patients were anastomosed with a 29‐mm stapler, and 28 female patients were anastomosed with a 31‐mm stapler. In addition, 36 male patients were anastomosed with a 29‐mm size stapler, and 60 male patients were anastomosed with a 31‐mm stapler. Significant use of the 29‐mm stapler among female patients and the 31‐mm stapler among male patients was observed.

The patient's ages ranged from 23 to 83 years, with a mean of 55.93 years. The mean age for a stapler size 29 mm was 59.61, and the mean age for a stapler size 31 mm was 52.25. No age‐related difference in stapler size was observed. (Table 1).

**Table 1:** Baseline characteristics of study participants.

Characteristics	Value^a^
Age (year)	55.93 ± 14.17
Gender	
Male	115 (63.9%)
Female	65 (36.1%)
Anastomotic Stricture	6 (3.3%)
Stapler size (mm)	
29‐mm	90 (50%)
31‐mm	90 (50%)

^a^ Data reported as number (percentage) and mean ± standard deviation, where applicable.

Follow‐up rectosigmoidoscopy revealed an anastomotic stricture in 6 (3.3%) patients. Regarding anastomotic stricture, there were no significant differences between cohorts based on stapler size (*p* = 0.682). All patients were successfully treated with endoscopic dilation. (Tables 2 and 3).

**Table 2:** Characteristics of study participants stratified by stapler size.

Characteristics	29‐mm stapler (*N* = 77)	31‐mm stapler (*N* = 96)	*p*‐value^a^
Age (year)	55.88 ± 14.82	55.98 ± 13.57	0.962
Gender count (male)	40 (52.2%)	52 (54.1%)	0.771
Body mass index (BMI)	24.63 ± 3.45	24.26 ± 3.78	0.507
Smoking	13 (16.8%)	15 (15.6%)	0.823
Diabetes mellitus	18 (23.3%)	19 (19.7%)	0.567
Ischemic heart diseases	4 (5.19%)	6 (6.20%)	0.765
Anastomotic distance from anal verge (cm)	8.63 ± 2.53	8.13 ± 2.96	0.238
Anastomotic Stricture	2 (2.2%)	4 (4.4%)	0.575
Anastomosis leakage	3 (3.8%)	5 (5.2%)	0.682

*Note*: Data reported as number (percentage) and mean ± standard deviation, where applicable.

^a^ *P*‐values were obtained from independent sample *t*‐test and chi‐square test, where applicable.

**Table 3:** Characteristics of study participants stratified by presence of anastomotic stricture.

Characteristics	Anastomotic stricture		*p*‐value^a^
	Present	Absent	
	(*N* = 6)	(*N* = 167)	
Age (year)	56.01 ± 14.09	53.50 ± 17.60	0.671
Gender (Male)	110 (63.2%)	5 (83.3%)	0.421

*Note*: Data reported as number (percentage) and mean ± standard deviation, where applicable.

^a^ *P*‐values were obtained from independent sample *t*‐test and chi‐square test, where applicable.

## 4 DISCUSSION

This study compared 29‐ and 31‐mm circular stapler size usage in rectal adenocarcinoma patients undergoing low anterior resection (LAR) at Imam Khomeini Hospital Complex, Tehran, Iran, between 2017 and 2021. The 29‐ and 31‐mm stapler size cohorts were comparable in terms of age and gender. The incidence of anastomotic stricture was compared between the two cohorts. The current study found no significant differences in the incidence of anastomotic stricture between the 29‐ and 31‐mm stapler cohorts. Consequently, our findings suggest that stricture‐related anastomoses are performed safely in both stapler‐size cohorts of rectal cancer patients undergoing LAR.

Prior studies have investigated the association between stapler size and the development of anastomotic strictures. A previous systematic review and meta‐analysis investigated the effect of circular stapler size on stricture rates following gastrointestinal anastomoses.^13^ Studies on oesophagal and gastric anastomoses suggest that a larger circular stapler is associated with a reduced risk of anastomotic stricture.^12^, ^13^ Moreover, studies on the IPAA have revealed that smaller stapler sizes are associated with a higher but insignificant stricture rate. In rectal cancer surgery, these studies are limited, yielding inconsistent results.^7^, ^8^, ^9^, ^11^

The results of this study corroborate findings from previous studies indicating that even though there is a correlation between stapler size and stricture in upper gastrointestinal tract, there is no correlation in lower GI.^1^, ^3^, ^8^, ^9^, ^14^, ^15^ Nagaoka et al. compared the safety of 25‐mm and 28/29‐mm circular staplers in sigmoid and rectal cancer patients undergoing curative colorectal resection with DST anastomosis. Patient groups were compared regarding the incidence of anastomotic complications, including strictures. The study observed no differences in anastomotic complications and strictures between the 25‐ and 28/29‐mm staplers. Diverting stoma and anastomotic leakage, as opposed to the size of the circular stapler, were risk factors for anastomotic strictures.^8^

Bertocchi et al. studied the risk factors for anastomotic stricture in patients undergoing rectosigmoid resection for deep infiltrating endometriosis. The existence of an ileostomy and prior pelvic surgery were risk factors for developing symptomatic anastomotic stricture. Comparing 29‐ and 31‐mm circular staplers revealed that stapler size was not a significant predictor of stricture development.^15^ Hu et al.^9^ and Polese et al.^3^ explored the risk factors for colorectal anastomotic stricture. They observed no significant associations between stapler size and anastomotic strictures.

However, our findings do not support the conclusions of several studies examining the relationship between stapler size and anastomotic strictures in colorectal surgery.^7^, ^11^, ^16^ Reif de Paula et al. studied the effect of stapler size on the risk of anastomotic complications in elective left‐sided colorectal resections. They included patients undergoing various surgical procedures (low anterior resection or proctosigmoidectomy, segmental, sigmoid, or left colectomies, and total or subtotal colectomies) for various indications (low anterior resection or proctosigmoidectomy, segmental, sigmoid, or left colectomies, and total or sub (colorectal cancer, diverticular disease, inflammatory bowel disease, chronic constipation, and others). The anastomotic strictures were defined as clinically significant changes proven on radiologic or endoscopic evaluations. They observed that the 25–29‐mm staplers were associated with a higher rate of anastomotic stricture than the 30–33‐mm staplers (7.1% vs. 2.1%; *p* = 0.007).^11^

Sandilos et al. studied patients following colorectal resection for various indications (neoplastic lesions, diverticulitis, and endoscopically unresectable polyps, among others) in elective, urgent, or emergent settings to determine the incidence of colorectal anastomotic strictures and associated risk factors. Among 141 patients, 20 (14.1%) developed strictures that were endoscopically detected at 19 weeks postoperatively. Anastomoses performed with a 28/29‐mm circular stapler had a significantly higher stricture rate than a 31/33‐mm stapler (*p* = 0.045).^7^

The heterogeneity of the study participants in terms of the various surgical indications, surgical procedures, and approaches, as well as the absence of a universal definition for anastomotic strictures, can partially explain the discrepancy between our findings and those of the studies above.

### 4.1 Limitations

We acknowledge the limitations of our research. The study's primary limitation was that it was conducted in a single center with small sample size. Selection bias is another potential concern due to the surgeon's discretion in selecting the stapler size, which was not controlled in the retrospective design of our study. Several variables, including tumor distance from the anal verge, which was not included in our analysis, could contribute to developing strictures or act as confounding factors. In addition, we exclusively studied patients with a diverting stoma. Consequently, we cannot confidently generalize the findings of our study to non‐diverted patients.

Despite its limitations, this study was conducted exclusively on rectal cancer patients undergoing LAR with protective loop ileostomy, which was performed using double stapling (DST). The surgical indication was similar for all patients (rectal adenocarcinoma). Inferior mesenteric artery, vein ligation, and splenic flexure mobilization were performed in all patients using the same technique. Excluded from the study were patients with anastomotic leakage or tumor recurrence as potential contributors to developing anastomotic strictures.

## 5 CONCLUSIONS

This study examined the relationship between circular stapler size and anastomotic stricture in rectal cancer patients undergoing LAR. This study observed no significant differences in strictures between the 29 and 31 mm stapler cohorts. The results of this study support the notion that concerning strictures, the anastomoses can safely be performed using both staplers in rectal cancer patients undergoing LAR.

## AUTHOR CONTRIBUTIONS

**Behnam behboudi**: Conceptualization; Data curation; Formal analysis; Investigation; Resources; Writing—original draft; Writing—review & editing. **Seyed‐Mohsen Ahmadi‐Tafti**: Conceptualization; Formal analysis; Investigation; Writing—original draft. **Seyyed‐Alireza**: Data curation; Investigation; Resources; Writing—original draft. **Kiana Tadbir‐Vajargah**: Investigation; Writing—original draft. **Mohammad Sadegh Fazeli**: Conceptualization; Investigation; Supervision; Writing—review & editing. **Alireza Hadizadeh**: Conceptualization; Data curation; Investigation; Writing—original draft; Writing—review & editing. **Amirhossein Poopak**: Investigation; Resources; Visualization. **Mohammad Reza Keramati**: Conceptualization; Formal analysis; Investigation. **Alireza Kazemeini**: Conceptualization; Data curation; Formal analysis; Investigation; Visualization; Writing—original draft; Writing—review & editing. **Aryan Ayati**: Conceptualization; Formal analysis; Investigation. **Hannaneh Yousefi‐Koma**: Writing—original draft.

## CONFLICT OF INTERESTS STATEMENT

The authors declare no conflict of interest.

## ETHICAL APPROVAL

This study was approved by the Research Deputy and the Ethics Committee of the Tehran University of Medical Sciences (Reference number: IR.TUMS.IKHC.REC.1398.164) and was carried out per the ethical standards outlined in the 1964 Declaration of Helsinki and all subsequent revisions. All participants signed a written informed consent form.

## TRANSPARENCY STATEMENT

The lead author Alireza Kazemeini affirms that this manuscript is an honest, accurate, and transparent account of the study being reported; that no important aspects of the study have been omitted; and that any discrepancies from the study as planned (and, if relevant, registered) have been explained.

## Data Availability

Data are available on request due to privacy/ethical restrictions.
